# Yellow fever virus envelope protein expressed in insect cells is capable of syncytium formation in lepidopteran cells and could be used for immunodetection of YFV in human sera

**DOI:** 10.1186/1743-422X-8-261

**Published:** 2011-05-27

**Authors:** Maria CES Barros, Tatiane GCM Galasso, Antônio JM Chaib, Nicolas Degallier, Tatsuya Nagata, Bergmann M Ribeiro

**Affiliations:** 1 Cell Biology Department, University of Brasília, Brasília, DF, CEP 70910-970, Brazil; 2 Laboratório Central do Distrito Federal - LACEN-DF, Brasília, DF, CEP 70830-010, Brazil; 3 Institut de Recherches pour le Développement UMR182, LOCEAN-IPSL UPMC, 4 pl. Jussieu, case 100, Paris, 75252 Cedex 05, France

## Abstract

**Background:**

Yellow fever is an haemorrhagic disease caused by a virus that belongs to the genus Flavivirus (Flaviviridae family) and is transmitted by mosquitoes. Among the viral proteins, the envelope protein (E) is the most studied one, due to its high antigenic potencial. Baculovirus are one of the most popular and efficient eukaryotic expression system. In this study a recombinant baculovirus (vSynYFE) containing the envelope gene (*env*) of the 17D vaccine strain of yellow fever virus was constructed and the recombinant protein antigenicity was tested.

**Results:**

Insect cells infected with vSynYFE showed syncytium formation, which is a cytopathic effect characteristic of flavivirus infection and expressed a polypeptide of around 54 kDa, which corresponds to the expected size of the recombinant E protein. Furthermore, the recombinant E protein expression was also confirmed by fluorescence microscopy of vSynYFE-infected insect cells. Total vSynYFE-infected insect extracts used as antigens detected the presence of antibodies for yellow fever virus in human sera derived from yellow fever-infected patients in an immunoassay and did not cross react with sera from dengue virus-infected patients.

**Conclusions:**

The E protein expressed by the recombinant baculovirus in insect cells is antigenically similar to the wild protein and it may be useful for different medical applications, from improved diagnosis of the disease to source of antigens for the development of a subunit vaccine.

## Background

Yellow fever (YF) is an haemorragic disease caused by a virus and transmitted by mosquitoes through two distinct cycles: the urban YF, transmitted by *Aedes aegypti *and the sylvatic YF, maintained in a enzootic cycle by *Haemagogus *and *Sabethes *mosquitoes with monkeys as main hosts [1]. No cases of urban YF have been reported in Brazil since 1942 [2]. The sylvatic YF is mostly restricted to wild and rural areas but recent outbreaks amongst human visitors and travelers together with the reinfestation of urban areas with the vector mosquito *Aedes aegypti *have concerned health authorities about the reurbanization of YF.

The main mechanisms for YF control consists of vaccination and insect vector control in urban areas. Yet, in 2008, 228 YF epizootic cases were reported and 64 cases of dead monkeys ocurred just in January. These are more than the 104 YF epizootic cases and the 17 cases of dead monkeys during the whole year of 2007. Until July of the same year, 45 cases of YF amongst humans were confirmed with 25 deaths, which represents 55,6% case fatality rate [3]. Similar to what happens to other flavivirus diseases, the investment on improved diagnosis techniques for YF with faster and precise methods is crucial for the early detection and correct identification of yellow fever infection for prevention and control of disease spread by public health authorities besides correct epidemiological reports [4].

Although clinical diagnosis is sufficient during an epidemic, laboratory diagnosis is the definite method to confirm yellow fever infection mainly in sporadic cases because yellow fever main symptoms may be confused with a broad range of related diseases that vary from severe malaria to dengue or leptospirosis. Viral isolation in mosquito cells cultures is a sensitive technique for the first days of infection, during the viremic period of the disease, but currently yellow fever diagnosis is based on serology, mainly enzyme-linked immunosorbent assays (ELISA) [5]. Yet, serological tests still use the whole virus as the antigen pool which could be improved with specificity by the use of antigenic parts of the virus, minimizing the risk of cross reactions with other flavivirus and risk of infection by health professionals.

*Yellow fever virus *(YFV) is an enveloped virus with a positive sense, single stranded RNA genome of 10,862 bases coding for a single ORF of 10,233 bp. This ORF encodes three structural proteins (Capsid, pM, and E) and seven non-structural proteins (NS1, NS2a, NS2b, NS3, NS4a, NS4b, NS5) [6]. Among the viral proteins, the E protein is the most studied one, due to its high antigenic potencial.

E protein is involved in many events, such as receptor binding site for viral attachment [7], fusion, penetration, hemagglutination, host range and cell tropism [8]. It also has an important role in immunological anti-virus response, eliciting neutralizing antibodies and inducing protective response [9].

Native E protein presents itself as homodimers and its activity is intimately linked to its structure that suffers conformation rearrangements changing the native homodimer into a fusogenic homotrimer after entering cells by receptor-mediated endocytosis [7]. The conformational change occurs in the lower pH environment of the endosome where viral lipid envelope fusion with endossomal membrane, releasing the nucleocapsids into the cells cytoplasm [10]. Each E protein monomer has a molecular mass of 50-55 kDa and presents 3 distinct domains: domain I, II and III. Domain III is the immunoglobulin-like receptor binding domain [9] and is recognized by virus-neutralizing antibodies, being considered a target for diagnosis assays [8].

On the purpose of isolating viral parts and expressing them separately, different heterologous expression systems may be used and within these, the Baculovirus Expression System is one of the most popular and efficient.

Baculoviruses are large (30-60 × 250-300 nm), rod-shaped, double-stranded DNA (80-180 Kbp) viruses that are highly specific and only capable of replication in arthropod hosts [11]. This is why baculoviruses were first studied as biological control agents to protect crops and forests [12]. The *Autographa californica multicapsid nucleopolyhedrovirus *(AcMNPV) is the most studied baculovirus at the molecular level and most expression vectors are based on this virus [13]. There are many advantages of using the Baculovirus Expression System such as high expression levels and post-translational modifications that allows the expressed heterologous proteins to be correctly folded and biologically active [14].

In order to evaluate the potencial antigenic activity of the Yellow fever E protein, in this study, we constructed a recombinant baculovirus containing a cDNA encoding the *env *gene of yellow fever virus. This recombinant virus was used to infect insect cells and larvae of susceptible insects and the protein produced was tested for its antigenicity.

## Methods

### Virus and cells

*Autographa californica multiple nucleopolyhedrovirus *(AcMNPV), *Anticarsia gemmatalis multiple nucleopolyhedrovirus *(AgMNPV), and the recombinant viruses vSynScathL [15] and vSynYFE (constructed in this work) were propagated in the *Trichoplusia ni *cell line BTI-Tn5B1-4 (Tn5B) [16]. Tn5B cells were maintained in TC100 medium (Invitrogen) supplemented with 10% fetal bovine serum.

### Envelope gene amplification and cloning

Standard molecular cloning techniques were used as described in Sambrook et al. [17]. The DNA sequence encoding YFV *env *gene was isolated by RT-PCR. The RNA was extracted with TRIzol reagent (Invitrogen) from a sample of lyophilized suckling mice intracerebral tissue inoculated with YFV (kindly supplied by Evandro Chagas Institute, Brazil) and the complementar DNA (cDNA) was synthetized with ImPromII reverse transcriptase system (Promega) using only the reverse oligonucleotide YFE2460R (5'-CAGATCTCCTTAATCCGCCCCAACTCC-3') which anneals downstream of the *env *gene. This cDNA was used in a PCR reaction using the specific oligonucleotides YFE878F (5'-GTGACAGATCTGACCATTGCC-3') and YFE2460R (5'-CAGATCTCCTTAATCCGCCCCAACTCC-3') located upstream and downstream of the *env *gene. The oligonucleotides were designed to incorporate the N and C-terminal hydrophobic regions of the E protein responsible for the ER translocation (amino acids 271 to 285) and membrane anchoring properties (amino acids 740 to 778), respectively [18]. The PCR program used was 94°C/1 min; (94°C/1 min, 50°C/2 min, 72°C/3 min) × 35; 72°C/10 min. An expected 1,590 bp PCR fragment corresponding to *env *gene was amplified and cloned into the pGEM-T®-Easy vector (Promega), following the manufacturer's instructions. This recombinant plasmid was designated pGEMYFE and utilized to amplify the envelope gene in *E. coli *DH5-α cells (Invitrogen).

### Construction of the transfer vector pSynYFE

The 1,590 bp DNA fragment corresponding to the *env *gene was obtained by *Eco*RI digestion of the pGEMYFE DNA which was separated by electrophoresis in an agarose gel (0.8%) and the fragment corresponding to the *env *gene was purified from the gel using the GFX DNA extraction kit, according to the manufacturer's instructions (GE Healthcare Life Science). The purified fragment was then ligated with the pSynXIV VI+X3 plasmid [19] previously linearized with *Eco*RI, resulting in the recombinant plasmid pSynYFE. This plasmid has the *env *gene under the control of two promoters in tandem (pSyn and pXIV). The pSyn/pXIV-env gene cassette region is flanked by baculovirus homologous regions that permit allelic replacement into the baculovirus genome during the co-transfection of insect cells with DNA from pSynYFE and the baculovirus vSynVI^-^gal, a recombinant AcMNPV with the *lacZ *gene from *E. coli *inserted into the polyhedrin gene (*polh*) locus of AcMNPV [19].

### Isolation of the recombinant virus vSynYFE

Tn5B cells (10^6^) were co-transfected, using liposomes (CellFectin, Invitrogen), with the DNA (1 μg) from the plasmid pSynYFE and DNA (0,5 μg) from vSynVI^-^gal virus digested with *Bsu*36I, following the manufacturer's instructions. The viral DNA digestion with *Bsu*36I facilitates the isolation of recombinant viruses since only viral DNA that recombines with the pSynYFE plasmid become circular again and only the circular baculovirus genome is infective to insect cells. After 3 h post-transfection (h.p.t.), the transfection medium was removed and TC-100 medium supplemented with 10% fetal bovine serum was added. Recombination between homologous regions of viral genomic DNA of vSynVI^-^gal and viral sequences present in pSynYFE DNA resulted in the formation of the recombinant virus vSynYFE. These cells were maintained at 27°C for 7 days and the recombinant virus was purified by the end point dilution method using 96 well plates [13]. To confirm the presence of the *env *gene into the recombinant virus, Tn5B cells were infected with vSynYFE and maintained at 27°C for 96 h. Supernatant from the infected cells were used for purification of the recombinant virus by ultracentrifugation through a sucrose cushion, followed by resuspension of the virus particles (budded viruses) pellet in virus disruption buffer following the protocol described in O'Reilly et al. [13]. The viral DNA was purified from disrupted virions as described in O'Reilly et al. [13], and used to perform a PCR reaction with the specific *env *oligonucleotides YFE878F and YFE2460R as described above.

### Heterologous protein expression analysis

Tn5B cells were seeded at 1 × 10^6 ^cells per well in six well plates (TPP), mock infected and infected with wild type AcMNPV, vSynVI^-^gal, *Anticarsia gemmatalis multiple nucleopolyhedrovirus *(AgMNPV), and the recombinants vSynYFE and vSynScathL. Cells were harvested at 96 h.p.i. and centrifuged at 750 × g for 7 min. The pellet was washed in PBS buffer (137 mM NaCl, 2.7 mM KCl, 10 mM Na_2_HPO_4_, 2 mM KH_2_PO_4_, pH 7.4) and aliquots from the samples were then analysed in a 12% SDS-PAGE gel [20] using a Mini Protean Tetra Cell apparatus (BioRad) following the manufacturer's instructions. After electrophoresis, the gel was stained in a mixture of acetic acid:methanol:water (10:40:50) containing 0,1% Coomassie brilliant blue and destained afterwards in boiling water.

### Fluorescence analysis

Tn5B cell monolayers (1 × 10^6 ^cells), grown over glass laminules placed inside sixty millimeter plates, were infected with vSynYFE (10 pfu/cell) or mock-infected (negative control). At 72 h.p.i, the laminules containing the cell monolayers were used for immunostaining. Cells were fixed with ice cold acetone for one hour (-20°C) and then incubated for 30 min with a PBS/BSA 10% solution at room temperature. The laminules containing cells were then incubated for 2 h with an E specific monoclonal antibody [21] which reacts with the envelope protein of the wild (Asibi) and vaccine strains of yellow fever virus with no cross reaction against other flavivirus or for 30 min with a polyclonal antibody for detection of flavivirus (anti-FLAV fluorescein isothiocyanate-conjugated antibody), currently used in a public diagnosis laboratory of reference (LACEN). The laminules incubated with the monoclonal antibody were washed 3 times with PBS (pH 7.4) and stained with DAPI (4,6-diamidino-2-phenylindole). Cells were again washed 3 times with PBS and incubated with the Alexa488-conjugated anti-mouse antibody (Invitrogen). Finally, cells were embedded with 1 drop of A31632 Component B Image iT TM Fx Signal Enhancer (Invitrogen) and 1 drop of N-Propyl gallate (0,2 g in 4 mL of PBS with glycerol-1 mL) and observed with a fluorescence (Axiophot, Zeiss) and/or confocal (The Broadband Confocal Leica TCS SP5, Leica DMI Series EL6000) microscopes with excitation and emission settings appropriate for the dyes used.

### Structural and ultrastructural analyses of infected cells

Sixty millimeter plates (TPP) were seeded with Tn5B cells (1 × 10^6^) and infected with vSynYFE (10 pfu/cell). Mock infected cells and vSynYFE infected cells were observed 72 h.p.i and photographed in a light Axiophot microscope (Zeiss) and processed for electron microscopy. In brief, samples were fixed for 30 minutes (2% glutaraldehyde, 2% paraformaldehyde in 0.1 M sodium cacodylate buffer pH 7.4 with 5% sucrose), centrifuged at 750×g for 5 min, the pellet washed in the same buffer, post-fixed (1% osmium tetroxide, 0.8% potassium ferricyanide in the same buffer), contrasted in block with 0.5% uranyl acetate, dehydrated in acetone, and embedded in Spurr's resin. The ultrathin sections were contrasted with uranyl acetate/lead citrate and observed in a TEM JEOL 100C and JEOL 1011 at 80 kV.

### MAC-ELISA Immunoassay

The antigenic properties of the recombinant yellow fever virus envelope protein present in vSynYFE-infected insect cells were tested in a MAC-ELISA modified assay [22]. In brief, Tn5B cells were seeded at 1 x10^6 ^cells per well in six well plates, mock infected and infected with wild type AcMNPV and the recombinant vSynYFE. Cells were harvested after 96 h.p.i and centrifuged at 750 × g for 7 min. The pellet was washed in PBS buffer and this procedure was repeated thrice. Cells were ressuspended in a solution of PBS and human serum and reserved for a later time.

Two hundred μL of serum from two patients positivily diagnosed for yellow fever were diluted 1:40 in PBS containing 0,5% BSA (bovine serum albumin). A volume of 50 μL of this sample was added to the wells and the plate was incubated for 1 hour in a moist chamber at 37°C. The plate was then washed five times with PBS. A volume of 50 μL of antigen was added to the plate. The different antigens used in this assay were described above and consists of the 3 insect cell extracts (mock-infected, AcMNPV-infected and vSynYFE-infected). The plate was incubated overnight in a moist chamber at 4°C and then washed for 5 times with PBS. The anti-FLAV peroxidase conjugated antibody (Monoclonal Antibody 6B6C-1 Mab, HRP Conjugated, currently used for diagnosis of Flavivirus infection at LACEN, Brasília, Brazil) was added to the wells diluted 1/3000 in PBS plus 20% human serum. The plate was incubated for 1 h in a moist chamber at 37°C and then washed for 5 times with PBS. Peroxidase substrate solution, (100 μL of H_2_O_2 _and 2,2' azino-bis 3-ethylbenzthiazoline-6-sulphonic acid) was added and after 30 minutes at 37°C the OD (492 nm) of each well was measured thrice using a plate reader. All samples with an optical density above 0.2, higher than the negative control, were considered positive.

The same experiment was repeated with a pool of sera from three patients positivily diagnosed for dengue fever (pool for dengue virus, DENV, sorotypes 1, 2 and 3, D1,D2,D3). Positive control was based in the reaction between the same pool of sera with a pool of DENV1, 2 and 3 (D1D2D3).

## Results

### Construction of the recombinant baculovirus vSynYFE

**Figure 1:**
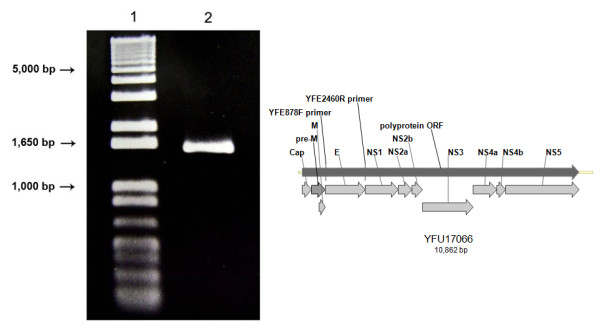
**Amplification of the envelope gene *(env)***. Amplification of the Yellow Fever *env *gene by RT-PCR. (A) Agarose gel (0.8%) eletrophoresis showing the RT-PCR amplification product (lane 2) using the oligonucleotides YFE2460R e YFE878F. Lane 1 shows the molecular mass marker, 1 Kb plus DNA ladder (Invitrogen). (B) Diagram of the Yellow Fever genome (Genebank accession # YFU17066 ) showing the main ORF (polyprotein ORF) and the position of the structural (Cap, pM and E) and non-structural (NS1, NS2a, NS2b, NS3, NS4a and NS5) proteins. The position of the oligonucleotides YFE2460R e YFE878F, used for the amplification reaction are also shown.

**Figure 2:**
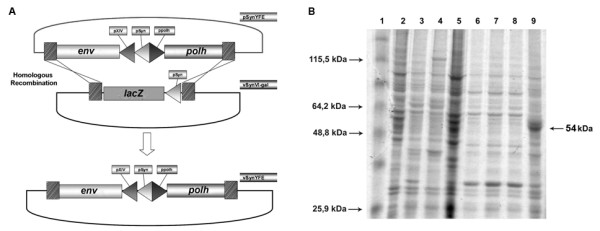
**Construction of recombinant virus and production of the E protein**. (A) Schematic representation of the construction of the recombinant virus vSynYFE, showing homologous recombination between the plasmid pSynYFE and the *lacZ *region (*polh *locus) in the virus vSynVI-gal. (B) SDS-PAGE analysis of E protein expression in insect cells. Lane 1, molecular weight marker (BenchMark™ Prestained Protein Ladder - Invitrogen); Lane 2, Tn5B cells mock infected; Lane 3, Tn5B cells infected with wild type AcMNPV; Lane 4, Tn5B cells infected with vSynVI^-^gal; Lane 5, Tn5B cells infected with *Anticarsia gemmatalis nucleopolyhedrovirus *(AgMNPV); Lanes 6, 7 and 8 Tn5B cells infected with a recombinant AcMNPV containing the *ScathL *gene from *Sarcophaga peregrina *(vSynScathL) and Lane 9, Tn5B cells infected with the recombinant vSynYFE. Arrow indicate a major polypeptide band of around 54 kDa in vSynYFE-infected Tn5B cells.

In order to construct the recombinant baculovirus vSynYFE, the DNA sequence encoding YFV envelope protein was isolated by RT-PCR from a sample of lyophilized suckling mice intracerebral tissue inoculated with YFV as a 1,590 bp band (Figure 1) and cloned (not shown) into the pGEM-T®-Easy vector (Promega). After transformation and amplification in *E. coli *(DH5-α, Invitrogen), the plasmid DNA was digested with *Eco*RI (data not shown) and the 1,590 bp fragment was cloned into the transfer vector pSynXIV VI+X3 generating the recombinat plasmid pSynYFE. The correct insertion of the *env *gene into the plasmid was confirmed by restriction enzyme digestion (data not shown). Finally, DNA from this plasmid was used to construct the vSynYFE recombinant baculovirus, by homologous recombination, after co-transfection with DNA from the vSynVI^-^gal virus inside insect cells (Figure 2a). To confirm the presence of the *env *gene into the recombinant virus, a PCR reaction was performed with viral DNA, released from the disrupted virions which amplified the expected 1,590 bp fragment (data not shown).

### Detection of the recombinant protein expression

Insect cells extracts infected with vSynYFE were used in a SDS-PAGE gel to detect envelope protein and, when compared to wild type infected cells, they presented a protein band of around 54 kDa (Figure 2b). This band was not present in Tn5B cells mock infected neither in cells infected with AcMNPV, AgMNPV and a recombinant AcMNPV containing the *ScathL *gene from *Sarcophaga peregrina *(vSynScathL) [15]. The 54 kDa recombinant protein produced has the expected size for the recombinant protein.

### Fluorescence analysis

**Figure 3:**
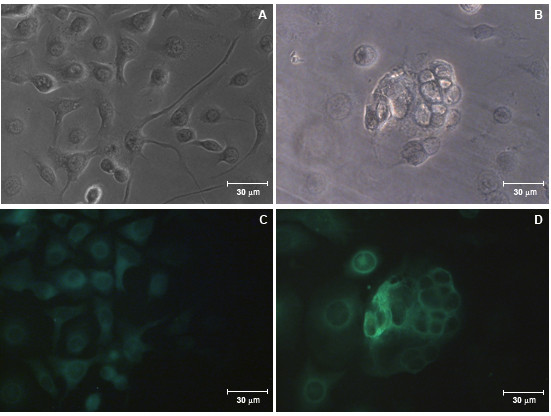
**E protein detection of vSynYFE-infected insect cells by fluorescence microscopy**. Tn5B cells were infected with vSynYFE (10 pfu/cell) and at 72 h p.i., cells were fixed with ice cold acetone and incubated with a polyclonal anti-FLAV antibody (conjugated with fluorescein isothiocyanate) and viewed in a fluorescence microscope. (A) Tn5B cells mock infected (bright field). (B) Tn5B cells infected with vSynYFE showing multinucleated syncytial cells (bright field). (C) Tn5B cells mock infected (fluorescence). (D) Tn5B cells infected with vSynYFE showing multinucleated syncytial cells (fluorescence). In A, B, C and D, Bars = 30 μm.

**Figure 4:**
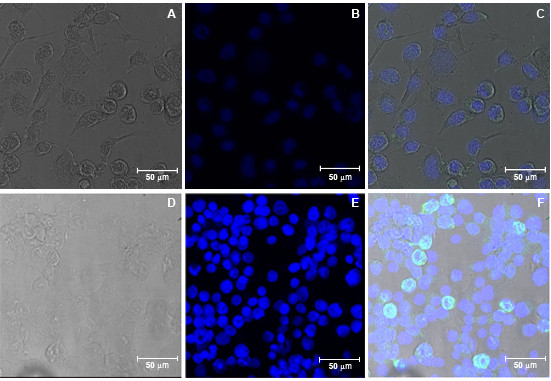
**E protein detection of vSynYFE-infected insect cells by confocal microscopy**. Tn5B cells were infected with vSynYFE (10 pfu/cell) and at 72 h p.i., cells were fixed with ice cold acetone and incubated with an E specific monoclonal antibody. Bound monoclonal anti-E antibodies were detected using Alexa488-conjugated anti-mouse antibodies by concofcal microscopy (green). Cell nuclei were visualized by DAPI staining (blue). (A) Tn5B cells mock infected (bright field). (B) Tn5B cells mock infected (DAPI staining). (C) Tn5B cells mock infected (merge). (D) Tn5B cells infected with vSynYFE (brightfield). (E) Tn5B cells infected with vSynYFE (DAPI). (F) Tn5B cells infected with vSynYFE (merge). In A, B, C, D, E and F, Bars = 50 μm. Blue staining (DAPI) indicates cells nuclei.

In order to confirm the imunogenicity of the E recombinant protein expressed in insect cells, fluorescence and confocal microscopy were performed. Polyclonal and monoclonal antibodies against the E protein were able to detect the presence of the recombinant E protein in the citoplasm of vSynYFE-infected Tn5B cells (Figure 3 and 4) indicating that the recombinant protein is antigenicaly similar to its authentic counterpart.

### Strutural and ultrastructural analysis of infected insect cells

**Figure 5:**
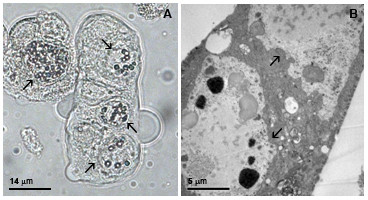
**E protein structural and ultrastructural analysis of vSynYFE-infected insect cells**. Tn5B cells were infected with vSynYFE (10 pfu/cell) and at 72 h p.i., the cells were photographed in a light microscope and processed for electon microscopy. Multinucleated syncytial Tn5B cells were easily observed by light (A) and electron microscopy (B). Bar in (A) = 14 μm and in (B), 5 μm. Arrows indicates cells nuclei.

Insect cells infected with vSynYFE showed multinucleated syncytial cells, which is a typical cytophatic effect of *Flavivirus *infection, which was not observed in Tn5B non-infected cells by light and electron microscopy (Figure 5).

### MAC ELISA Immunoassay

**Figure 6:**
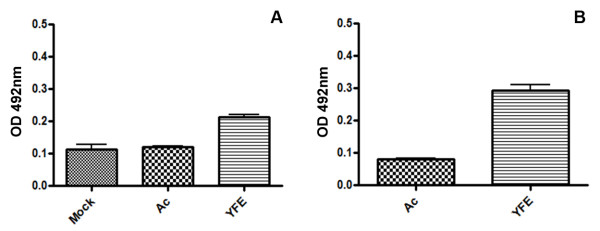
**Yellow fever MAC-ELISA assay**. In this modified IgM antibody capture ELISA (MAC-ELISA) the antigens tested were mock infected Tn5B cells, Tn5B cells infected with AcMNPV and Tn5B cells infected with vSynYFE. (A) and (B): Two different yellow fever-infected patients sera were used for the detection of antigens, they are known to react positively with yellow fever virus. The experiment was repeated 3 times.

**Figure 7:**
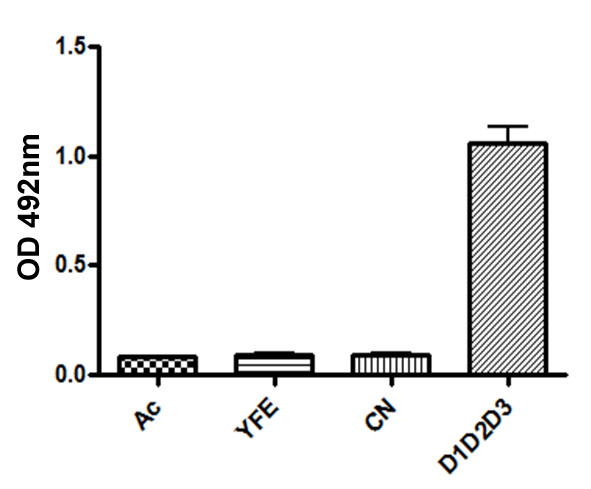
**Dengue MAC-ELISA assay**. In this modified IgM antibody capture ELISA (MAC-ELISA) assay a pool of sera from dengue virus-infected patients [containing antibodies against the 3 DENV sorotypes 1 (D1), 2 (D2) and 3 (D3)] were used with the following antigens: mock infected Tn5B cells, Tn5B cells infected with AcMNPV and Tn5B cells infected with vSynYFE. Positive control antigen is a pool of dengue virus (D1D2D3) and CN is the same assay with serum from a healthy individual. No reactivity was detected with vSynYFE-infected insect cells extracts treated with dengue-infected patients sera.

In this assay, we used two different yellow fever-infected patients sera for the reaction with mock-infected, AcMNPV- and vSynYFE-infected insect cells extracts as antigens. OD values shown in figure 6 are averages of the three repetitions of experiment measures and error bars were determined using Graph Pad Prism Software (Graphpad Software, San Diego,CA). Mock- and AcMNPV-infected cells extracts showed low OD values (0,113 and 0,121 respectively) when compared to the vSynYFE-infected insect cells (0,214) extracts (Figure 6A). AcMNPV-infected cells extracts also showed low OD values using the second yellow fever-infected patient sera (0,081) when compared to the vSynYFE-infected insect cells (0,294) extracts (Figure 6B). We also carried out a second assay with a pool of sera from dengue virus-infected patients and purified dengue virus as antigens (Figure 7). No cross reactivity was detected with the vSynYFE-infected insect cells extracts tested, indicating that vSynYFE-infected insect cells extracts could be used in substitution of the whole yellow fever virus as antigen in this assay.

## Discussion

In order to check the antigenic activity of a recombinant YFV E protein, we expressed this protein via a recombinant baculovirus (vSynYFE) in insect cells. Mammalian cells are very efficient to express E protein from yellow fever virus [23,24] and other flaviruses, such as DENV [25]. However, its use is much more expensive and cumbersome than insect cells which are also not infected by human pathogenic viruses [26]. Tn5B cells infected with vSynYFE expressed the predicted 54 kDa recombinant protein. The recombinant virus infected cells also showed the formation of multinucleated syncytial cells which is a typical cytophatic effect found in mosquito cells infected with Flavivirus [1]. In fact, this is one of the diagnosis techniques available for yellow fever. This infected cells were also positively immunostained with monoclonal and polyclonal antibodies raised against the E protein of the original *Yellow fever virus *and to flavivirus, respectively, and currently used in a diagnostic centre (LACEN) in the city of Brasília, Brazil. Furthermore, in a MAC ELISA assay, two different yellow fever-infected patients sera reacted positively with vSynYFE-infected insect cells extracts as antigens. This experiment would be more reliable if more patients sera were tested. Unfortunately, yellow fever positive serum is rare and most of it is distributed amongst reference centers of diagnosis and were not available. On the other hand, the recombinant virus infected insect cells extracts did not cross react with a pool of sera from dengue virus infected patients (Figure 7) which indicates that the recombinant envelope protein may respond antigenically very alike to what happens with the intact virus which is very relevant when considering that this might turn diagnosis tests more specific and less dubious. Cross reactivity with different Flavivirus is quite common during serological tests [27]. The use of recombinant proteins of the virus or even smaller parts of it, may diminish this kind of problem. It also could reduce costs too, since nowadays the use of the whole yellow fever virus as antigen for diagnosis is quite expensive since it requires a biosafety structure, with animal use and care.

Quicker diagnosis would mean quicker disease control. Subnotificated cases leads to delayed emergency response and the risk of reurbanization of the disease since the urban vector has adapted to living among humans in domestic environments. The use of recombinant envelope protein could be extended in the development of a safer vaccine which did not include the active virus at all. Especially when severe adverse events are being associated with the actual yellow fever vaccine with the attenuated virus, such as hypersensitivity reactions, associated neurotropic disease and associated viscerotropic disease [28].

Desprès et al. [29] used baculovirus to express and characterize yellow fever proteins E and NS1 (a non-structural yellow fever virus protein identified as the soluble complement-fixing antigen) and showed that insect cells infected with the recombinant baculoviruses synthesized E and NS1 proteins that were similar in size and antigenicity to those expressed in Vero cells infected with YFV. Their work resembled the methods used is this work but differed in the host cell type used, which were *S. frugiperda *insect cells, the vector used for the recombinant virus construction and the length of the E protein gene used. In their work, no syncytial formation in virus-infected *S. frugiperda *cells was reported. This could be explained by the expression of a truncated version of the E protein without the C-terminal hidrophobic region (amino acids 740 to 778) responsible for the transmembrane localization of the protein inside intracelular membranes which may be important for the oligomerization and folding of the E into a biological active protein [18].

The same research group injected recombinant virus-infected insect cell extracts into mice and conducted a fatal YFV challenge [29]. The result was a solid protection against lethal YFV encephalitis, which reinforce the recombinant protein antigenicity when compared to the whole virus. Shiu et al. [30], also expressed recombinant E protein in insect cells using recombinant baculovirus. The recombinant virus genome contained a cDNA coding for amino acids 109 to 820 of the YFV polyprotein. This region has a N-terminal truncated pM, the complete M and E proteins and the first 42 amino acids of the NS1 protein. This recombinant protein expressed in insect cells was shown to be antigenically indistinguishable from the E protein of yellow fever vaccine virus.

The actual yellow fever vaccine strain is considered to be very safe, in fact, its considered to be one of the safest vaccines ever developed [31]. Its production has been administered to man since the late 1930s [24] and a single dose of 17D yellow fever vaccine confers long-term immunity, with some data confirming cases exceeding the expected 10 years of protection [32]. However, as it still makes use of attenuated virus, some adverse events might result from its use [33] causing viscerotropic and neurotropic adverse events and anaphylaxis [34]. Yellow fever vaccine associated viscerotropic disease (YEL-AVD) resembles wild yellow fever symptoms with a similar fatality rate while yellow fever vaccine associated neurologic disease (YEL-AND) may manifest encephalitis, meningitis, neuropathy, Guilllain-Barre syndrome, acute disseminated encephalomyelitis or spinal myelitis [35]. Their ocurrence varies from 0,4 to 7,9 cases per 100.000 vaccine doses and some alternatives are being studied in order to minimize the risks of vaccine administration specially for the ones with a compromised immunologic system such as elderly people, immunosuppressed or thymectomized patients, infants under 9 months of age and pregnant or nursing women. One alternative would be the administration of inactivated virus which is already being tested in clinical trials [34]. Other would be to use viral-like particles as immunogens.

Viral-like-particles (VLP) produced in insect cells have been used in subunit vaccine trials for the influenza infection and preliminary studies demonstrated that this VLPs are capable of inducing immunity against some strains of influenza virus [36]. Gut-Winiarska et al. [37] developed a direct sandwich blocking ELISA diagnostic test using a recombinant glycoprotein B (gB) expressed in baculovirus to detect pseudorabies virus. It showed high sensitivity and specificity when compared to two other commercial tests using the whole virus. Recently, a baculovirus vector based vaccine confering protection against *Human papillomavirus *(HPV) (causative agent of cervical neoplasia) was approved for use in Brazil and the Brazilian Ministry of Health is considering to adopt it in its current annual vaccination program [38].

## Conclusions

In conclusion, our data shows that the recombinant E protein derived from YFV expressed in insect cells is recognized by monoclonal and polyclonal antibodies used to detect YFV infection in human sera and therefore, may be useful for different medical applications, from improved diagnosis of the diseases to source of antigens for the development of a subunit vaccine, which might be useful in the future to promote immunization of individuals contraindicated to receive the current yellow fever attenuated virus vaccine.

## Competing interests

The authors declare that they have no competing interests.

## Authors' contributions

MCESB and TGCMG carried out the study, performed analysis of data and drafted the manuscript. TN helped with the construction of recombinant viruses and with the confocal analysis of virus-infected cells. AJMC helped with the MAC ELISA assay protocol and provided lyophilized suckling mice intracerebral tissue inoculated with YFV. ND provided part of research funds and discussed partial results. BMR conceived the study, provided research funds, supervised students and revised the manuscript. All authors have read and approved the final manuscript.
